# Estimation of Horizontal Eddy Heat Flux in Upper Mixed-Layer in the South China Sea by Using Satellite Data

**DOI:** 10.1038/s41598-018-33803-2

**Published:** 2018-10-19

**Authors:** Jiayi Pan, Yujuan Sun

**Affiliations:** 1 School of Marine Sciences, Nanjing University of Information Science and Technology, Nanjing, Jiangsu China; 2 Institute of Space and Earth Information Science, The Chinese University of Hong Kong, Hong Kong, China; 3 Shenzhen Research Institute, The Chinese University of Hong Kong, Shenzhen, Guangdong, China; 4 Fisheries and Oceans Canada, Bedford Institute of Oceanography, Dartmouth, Nova Scotia Canada; 5 First Institute of Oceanography, State Oceanic Administration, Qingdao, Shandong China

## Abstract

In this study, the horizontal eddy heat flux in the upper mixed-layer in the South China Sea (SCS) is derived from satellite-derived observational data of sea surface height anomalies and optimally interpolated sea surface temperature, as well as a reanalysis dataset of mixed-layer depth. The long-term heat flux shows a northward transport on the west side of the SCS, comparable with that in the Kuroshio extension with strong eddy activities. The eddy flux in the SCS has a prominent semi-annual cycle and becomes the strongest in winter and summer with the inflow flux in the south and the outflow in the northwest into the East China Sea through the Taiwan Strait. The semi-annual cycle is related to the strong semi-annul variabilities of the velocity and the temperature in areas southeast of Vietnam and in the northern SCS, respectively. In some areas of the SCS, the eddy heat flux can reach more than ~ 60% of the mean flow heat flux. The convergence of the eddy flux indicates that heat accumulates southeast of Vietnam, which may result in heat storage increases in the upper mixed-layer.

## Introduction

The oceanic eddy kinetic energy may greatly affect time-varying general circulations everywhere in the world oceans^1,2^. The horizontal eddy heat flux is one of the important components in the global heat budget and plays a vital role in the upper ocean thermal dynamics. The meridional eddy heat transport can account for as much as 20% of the mean flux in some regions^1,3^. Strong eddy heat fluxes are typically found in western boundary current extensions where meso-scale eddies, meanders, and planetary waves are active^4,5^. Although the eddy heat flux is insignificant in the ocean interior, because of the large extent of the Antarctic Circumpolar Current, it can be a dominant factor in the meridional heat transport in the Southern Ocean^6^. It was found that internal changes in the equatorward transport of thermocline waters is closely related to eddy variability, implying an important mechanism in the air–sea and climate system^4^. Thus, accurate understanding of the eddy horizontal heat flux is one of the key factors that affect the efficiency of ocean and atmosphere system modeling systems^7,8^.

Oceanic eddy variability can be treated as turbulence in the geostrophic balance and supports energy cascades between different spatial scales^9,10^. It has been suggested that the covariance of temperature and velocity could be used to calculate the eddy heat transport^1^. Eddy fluxes are estimated using data acquired from moored current meters^11^. In recent years, satellite observations have been used to derive eddy temperature and heat fluxes^1,5,12–14^. In many studies, emphasis was placed on the western boundary current extensions with thermocline tilts in the zonal direction due to large scale baroclinic waves^4,5,11^. Other regions still lack sufficient investigations, though horizontal eddy fluxes exist in the most ocean areas where eddy variability is active^1^.

The South China Sea (SCS) is a semi-enclosed marginal sea connecting the open Pacific mainly through the deep-water Luzon Strait to the east, and also via a number of smaller and shallower passages in the Java Sea to the south. Eddy variability in the SCS was found to be highly active all year round^15–22^. Therefore, in the SCS, the eddy heat flux could be an important factor influencing the total heat budget^23^. It is found that the SCS upper layer heat structure can greatly affect the tracks and intensities of typhoons generated in and passing through the area. It is of great significance to clarify the horizontal heat transport in the SCS as well as the heat exchange with exterior basin (e.g., the Pacific). In this study, using satellite data of sea surface height and sea surface temperature, as well as mixed-layer depth reanalysis data, the eddy heat flux in the upper mixed-layer in the SCS is derived, and its seasonal variation is analyzed, along with heat flux convergence and divergence characteristics. The results help to better understand the heat forcing in the regional thermal dynamics, a key issue for the design of accurate numerical simulation and weather forecasting in the SCS.

## Data and Methods

The temperature flux can be derived by the eddy correlation, given by^1^:

1 $${F}_{T}^{x}=\overline{T^{\prime} u^{\prime} },$$

and

2 $${F}_{T}^{y}=\overline{T^{\prime} v^{\prime} }$$

where *F*_*T*_ represents the temperature flux and the superscripts, *x* and *y* denote flux components in zonal and meridional directions, respectively. The *u* and *v* are the zonal and meridional velocity components of the ocean current, respectively. The prime and overbar on the right-hand sides of Equations (1) and (2) represent time-variable components and the ensemble average, respectively. Assuming the heat content (or storage) in the upper mixed-layer is of more significance to the air-sea exchange, we can estimate the eddy heat flux in the upper mixed-layer using:

3 $${F}_{Q}^{x}=\rho {C}_{p}H\overline{T^{\prime} u^{\prime} }$$

and

4 $${F}_{Q}^{y}=\rho {C}_{p}H\overline{T^{\prime} v^{\prime} ,}$$

where *F*_*Q*_ is the horizontal eddy heat flux, *H* represents mixed-layer depth, and *C*_*p*_ is the specific heat capacity. For the geostrophic balance, Equations (3) and (4) can be rewritten as

5 $${F}_{Q}^{x}=-\frac{\rho g{C}_{p}H}{f}\overline{T^{\prime} \frac{\partial h^{\prime} }{\partial y}},$$

and

6 $${F}_{Q}^{y}=\frac{\rho g{C}_{p}H}{f}\overline{T\text{'}\frac{\partial h\text{'}}{\partial x}},$$

where *h* is the sea surface height, and the *h*′ represents the sea surface height anomaly.

In this study, sea surface height anomaly data measured by satellite altimeters are used to calculate $\partial h^{\prime} /\partial x$ and ${\rm{\partial }}h^{\prime} /{\rm{\partial }}y$. The data sets are the merged product derived from TOPEX/POSEIDON, Jason-1/2 (French-US altimeter satellites), ERS-1/2 (European Remote Sensing satellites), and ENVISAT (European Remote Sensing satellite) altimeter observations with a spatial grid of 1/3° by 1/3° available on the AVISO website (http://www.aviso.oceanobs.com/). The daily absolute geostrophic velocity data are also downloaded from the AVISO website, which are a gridded multi-mission altimeter product at the 1/3° by 1/3° spatial resolution, including global absolute dynamic topography.

The temperature data are from the daily Optimally Interpolated (OI) sea surface temperature (SST) product blending together both microwave (MW) and infrared data (IR). The data are enhanced with interpolation to fill the missing regions due to orbit transition and other environmental factors, achieving a resolution of ~9 km^24^.

The daily mixed-layer depth (MLD) data are obtained from the Mercator Ocean Global Ocean Physics Reanalysis, GLORYS2V4, available on the Copernicus Marine Environment Monitoring Service (CMEMS) website (http://marine.copernicus.eu). The GLORYS2V4 reanalysis is generated with the NEMOv3.1 ocean model, which assimilates along-track satellite altimetry, sea surface temperature, sea ice concentration, and *in-situ* profiles of temperature and salinity to provide datasets including daily temperature, salinity, currents, sea surface height, and sea ice parameters at ¼ degree horizontal resolution with 75 vertical levels. In the GLORYS2V4 reanalysis products, the mixed-layer depth is determined as the depth where the density increase compared to density at 10 m depth corresponds to a temperature decrease of 0.2 °C in local surface conditions^25^.

The density of ocean water at the sea surface is set as 1027 kg m^−3^, and the specific heat capacity of seawater, *C*_*p*_ is 3993 J kg^−1^ K^−1^. The study area is in 5~30 °N, and 99~125 °E. In this study, long-term means are calculated over the period from December 2005 to November 2010.

## Results

### Long-term means

**Figure 1:**
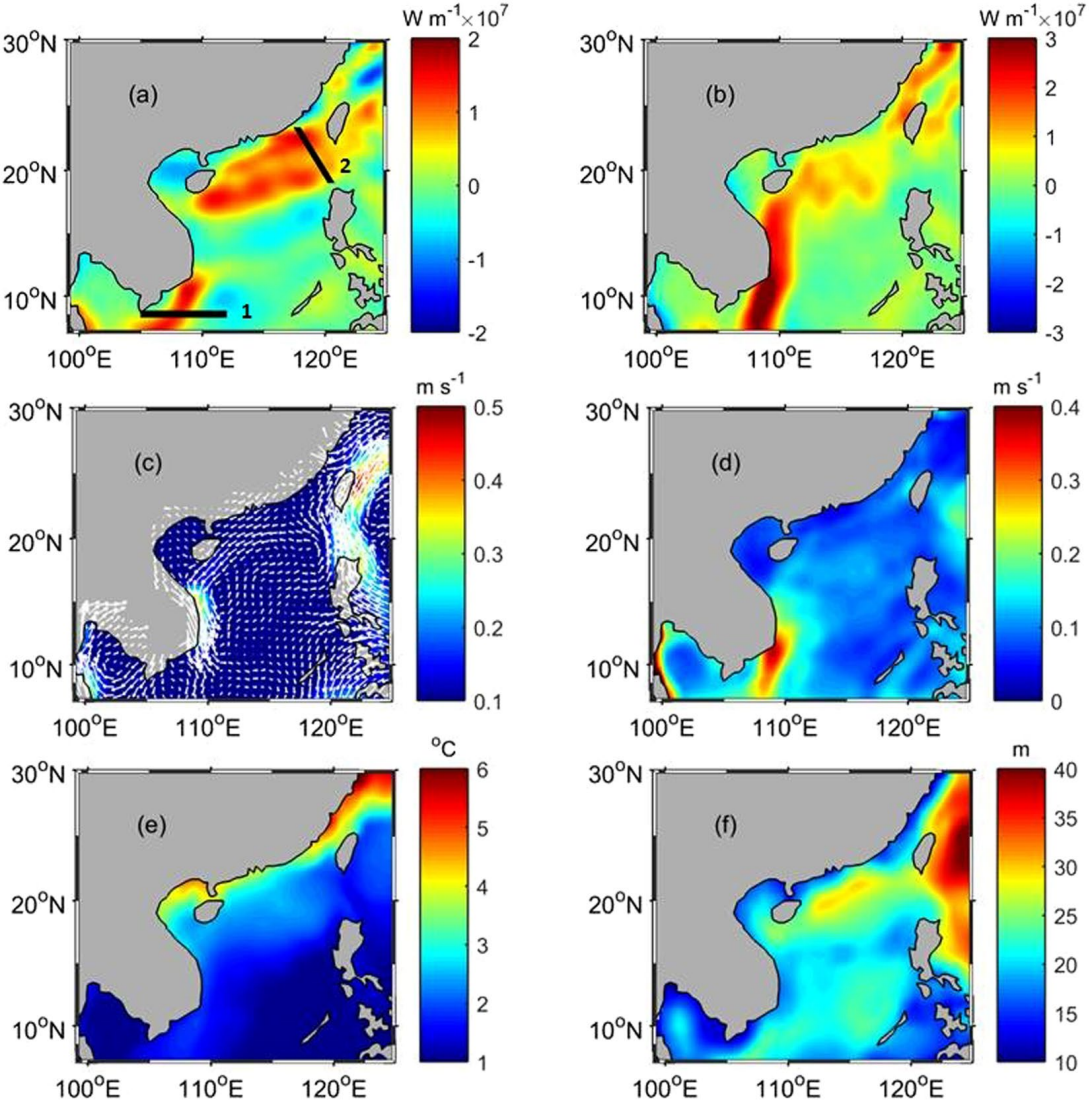
Long-term eddy heat flux for zonal (**a**) and meridional (**b**) components, the mean velocity (**c**), mean velocity variability (**d**), mean temperature variability (**e**), and mean mixed-layer depth (**f**). The maps in Fig. 1 are generated by using the Matlab software (http://www.mathworks.com/products/matlab/).

The 5-year mean of eddy heat flux in the upper mixed-layer in zonal (a) and meridional (b) directions is shown in Fig. 1. In the northern SCS (17.7~22.2 °N, 110~120 °E), the positive (eastward) eddy heat flux appears with the maximum around 1.5 × 10^7^ W m^−1^. In the central SCS between 14.0 and 17.5 °N is a region with a westward eddy heat flux, another westward eddy heat flux appears in the southern SCS between 109.0 and 112.0 °E. The spatial characteristic of the meridional eddy heat flux is much more distinct, with a high northward flux on the west side of the SCS. The maximum northward flux appears southeast of Vietnam around 9.53 °N, 108.68 °E with a value of 4.0 × 10^7^ W m^−1^. The amount is comparable to the eddy heat flux in the Kuroshio extension where the strong eddy activities exist^5^. In addition, the northward flux region is shown in the northern SCS, as well as in the coastal area of southeast China. Southward eddy heat fluxes exist in the southern SCS on the east side of the northward flux region. Although the southward flux balances a portion of the northward flux, the total meridional eddy heat is transported northward in the southern SCS. Generally, the eddy heat fluxes into the SCS from the tropical Pacific in the south through the Java Sea goes northward on the west side, and turns eastward in the north, and the heat flux is directed out of the SCS to the northeast through the Taiwan Strait and the Luzon Strait. The eddy heat transport is calculated along the two sections shown in Fig. 1a, one located southeast of Vietnam (section 1) and the other (section 2) in the northern SCS, where the high fluxes are found. For section 2 (in the northern SCS), the heat transport reaches 3.9 × 10^12^ W, and for section 1 (southeast of Vietnam), the transport is 10.5 × 10^12^ W, 2.7 times as high as that in the northern SCS section, suggesting that the eddy variability may transport more heat into the SCS than out.

Figure 1c–f illustrate the mean velocity, mean velocity variability (defined as $\overline{\sqrt{{u^{\prime} }^{2}+{v^{\prime} }^{2}}}$), temperature variability (defined as $\overline{\sqrt{T{\text{'}}^{2}}}$), and the mean mixed-layer depth ($\bar{H}$), respectively. By definition, the velocity variability represents the level of the mean eddy kinetic energy. The strong velocity variability (Fig. 1d) appears southeast of Vietnam, revealing that strong eddy kinetic energy and strong eddy heat flux co-exist in this region. However, the sea surface temperature variability (Fig. 1e) and mixed-layer depth (Fig. 1f) are both relatively low in this area. Thus, the strong eddy heat flux appearing southeast of Vietnam can be associated with the enhanced eddy variability. In the northern SCS, although the velocity variability is relatively weak (<0.1 m s^−1^) compared with that (~0.6 m s^−1^) southeast of Vietnam, the temperature variability (with a maximum of 4.7 °C) and the mixed-layer depth (25~30 m) are both high compared with those in other areas of the SCS; therefore, the strong heat flux in the upper mixed-layer in the northern SCS can be related with the large temperature fluctuations and the relatively deep mixed-layer depth. Figure 1c shows that the mean flow is enhanced east of Vietnam, which may cause strong variability there, as a result of frequently changing wind, flow instability, and the coastal morphology. Compared with the zonal component, the meridional velocity has much higher variability along the southward/southwestward current on the west side of the SCS. This high meridional velocity variability induces a strong northward eddy heat flux along the route of the western boundary current in the SCS. Figure 1c reveals that the western boundary current in the SCS flows southward, which may cause a southward net heat transport by the mean flow, while the eddy heat flux transports heat northward.

### Seasonal cycle

**Figure 2:**
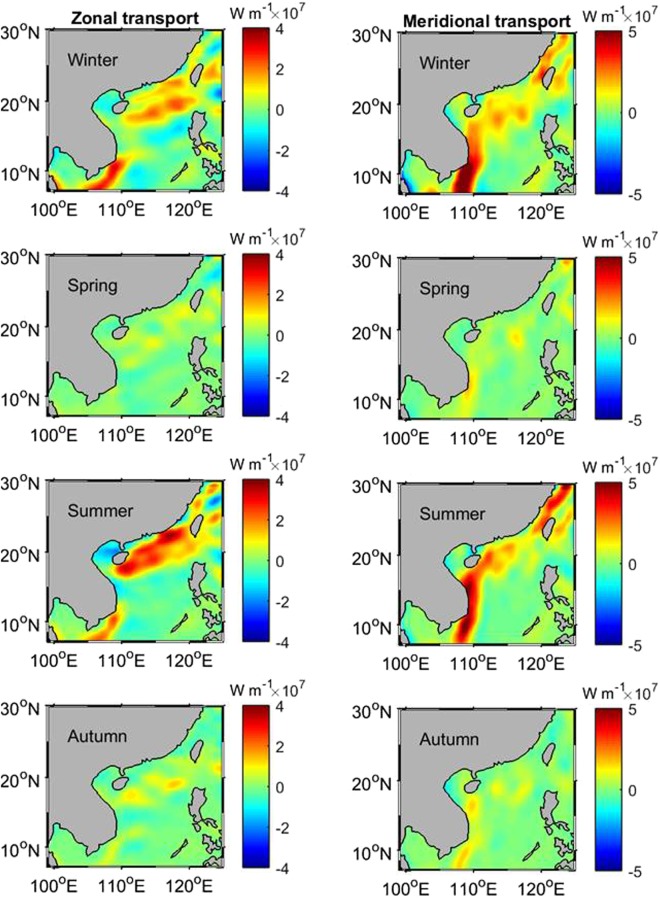
Eddy heat flux in winter, spring, summer and autumn for zonal (left panel) and meridional (right panel) components. The maps in Fig. 2 are generated by using the Matlab software (http://www.mathworks.com/products/matlab/).

In order to calculate the seasonal eddy heat flux, seasonal means of the mixed-layer depth are used in Equations (3 and 4), as ${F}_{Q}^{x}=\rho {C}_{p}{H}_{s}\overline{T^{\prime} u^{\prime} }$ and ${F}_{Q}^{y}=\rho {C}_{p}{H}_{s}\overline{T^{\prime} v^{\prime} }$, where *H*_*s*_ represents the seasonal mean of the mixed-layer depth which varies for the different seasons. The ensemble mean is calculated over a seasonal timespan (3 months), namely December-February for winter, March-May for spring, June-August for summer, and September-November for autumn. Figure 2 illustrates the eddy heat flux in winter, spring, summer, and autumn for the zonal (left panel) and meridional (right panel) components. The strongest eddy heat flux is seen in winter. The spatial pattern of the flux in winter is consistent with the long-term characteristics (Fig. 1a,b), with higher values of both positive and negative fluxes than the long-term. For the meridional component, the northward flux on the west side of the SCS is very strong, and heat is transported out of the SCS through the Taiwan Strait and the northern Luzon Strait through the eddy flux. The heat can be further fluxed into the coastal regions of southeast China in the East China Sea (ECS) through the Taiwan Strait. The SCS may act as an important channel for the eddy heat transport from the tropical Pacific to the ECS. In spring, the heat flux becomes weakest, and although a similar spatial pattern exists for the meridional component as seen in the winter, the zonal flux is insignificant.

The heat flux reaches another peak in summer, and the spatial pattern of the summer flux is consistent with that in winter, showing that the eddy heat energy transport from the tropical region to the mid-latitude exists even in the summer season. In autumn, the flux keeps a similar pattern, then weakens again, but is still stronger than in the spring. However, the along-shore flux off the coast of southeast China is not present.

**Figure 3:**
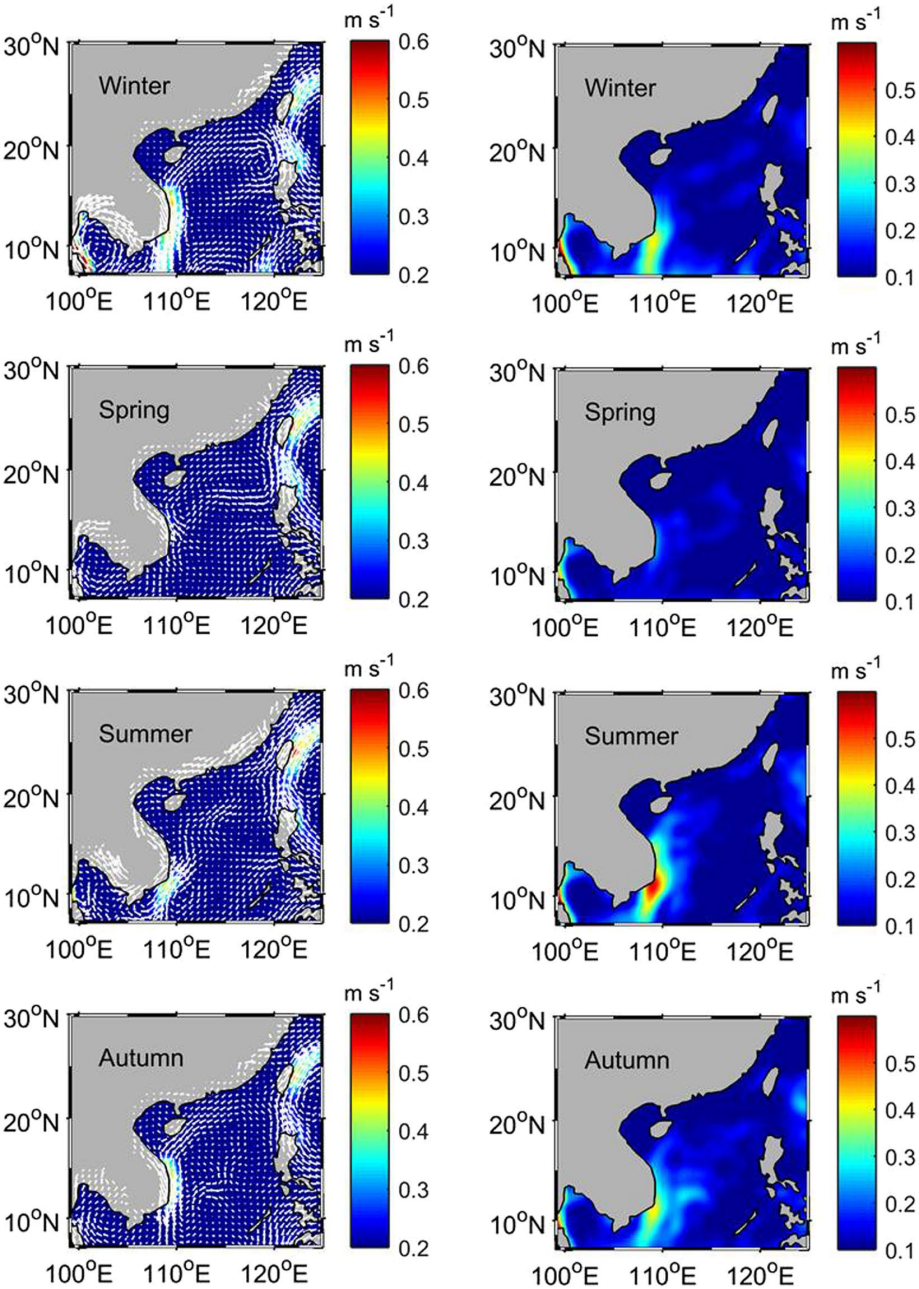
The seasonal means of the velocity (left panel) and velocity variability (right panel). The maps in Fig. 3 are generated by using the Matlab software (http://www.mathworks.com/products/matlab/).

**Figure 4:**
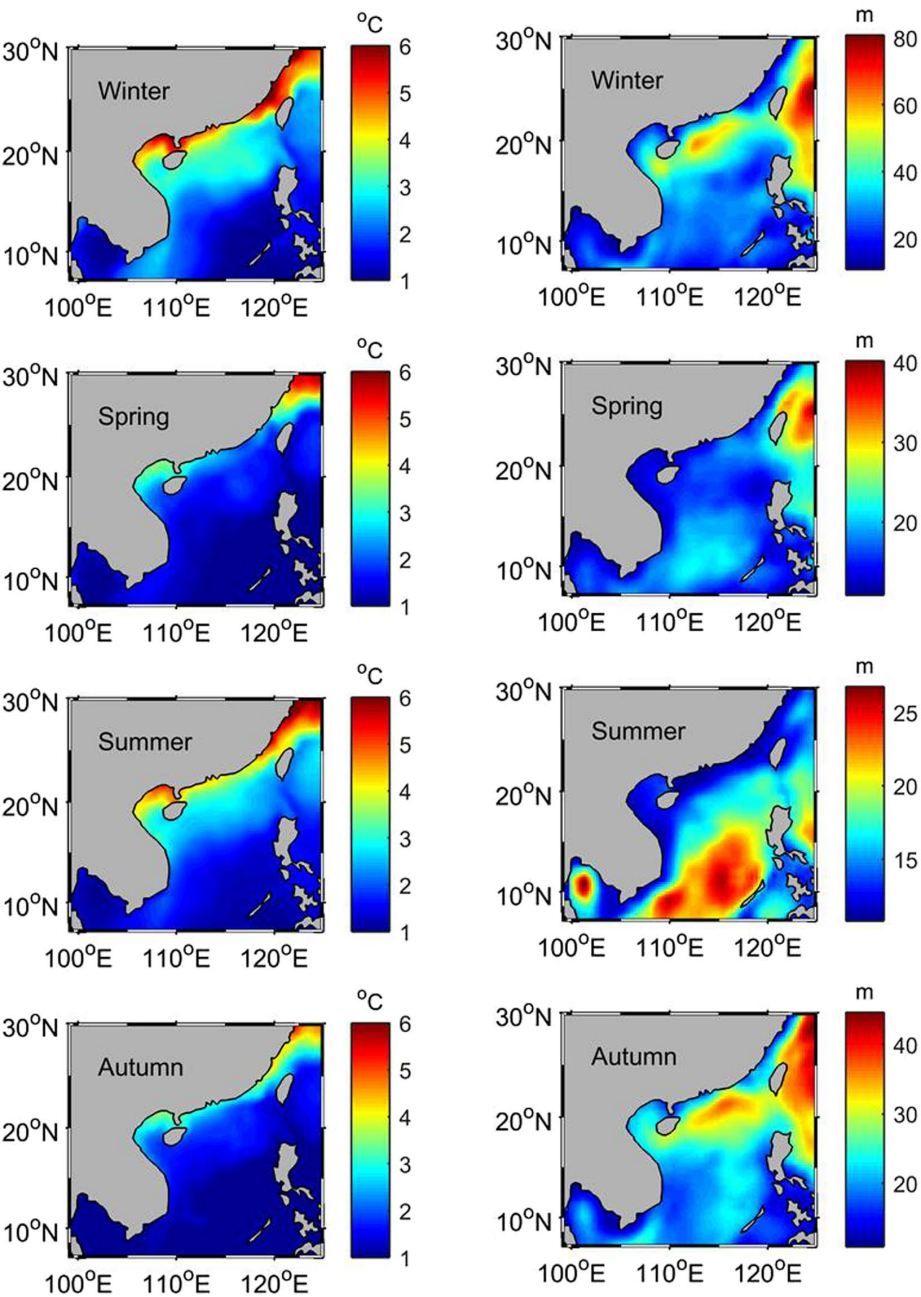
The seasonal means of sea surface temperature variability (left panel) and mixed-layer depth (right panel).The maps in Fig. 4 are generated by using the Matlab software (http://www.mathworks.com/products/matlab/).

Figure 2 reveals that there is a semi-annual cycle in the eddy heat flux, high in winter and summer and low in spring and autumn. To explore the mechanism of the semi-annual cycle, the seasonal means of the velocity, velocity variability ($\overline{\sqrt{{u^{\prime} }^{2}+{v^{\prime} }^{2}}}$), sea surface temperature variability ($\overline{\sqrt{T{\text{'}}^{2}}}$), and mixed-layer depth ($\bar{H}$) are calculated and shown in Fig. 3 (velocity in the left panel and its variability in the right panel) and 4 (temperature variability in the left panel and mean mixed-layer depth in the right panel). Influenced by monsoonal winds over the SCS, the mean flow switches the direction between the winter and summer with the southeastward current in winter and the northwestward current in summer, especially in the western SCS and the northern SCS (Fig. 3a vs. 3c). The right panel of Fig. 3 indicates that the velocity variability (representing the level of the eddy kinetic energy) has a strong semi-annual cycle southeast of Vietnam. In the northern SCS there is a semi-annual temperature variability, strong in winter and summer and weak in spring and autumn (left panel of Fig. 4), while the mixed-layer depth only exhibits an annual cycle (right panel of Fig. 4). This suggests that the semi-annual cycle of the eddy heat flux southeast of Vietnam is associated with the velocity fluctuations, and in the northern SCS, temperature variability is the causative factor for the semi-annual cycle in the eddy heat flux. Forced by the strong monsoonal winds, the mean flow becomes the strongest in winter and summer, and consequently, a maximum velocity variability is seen in both summer and winter.

## Discussion

**Figure 5:**
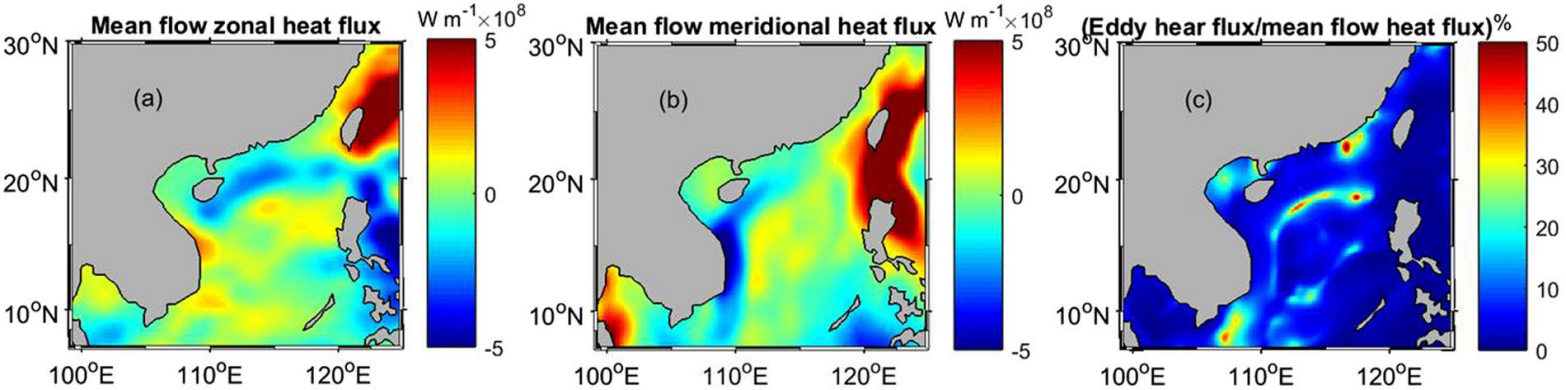
Long-term of mean flow-caused heat flux for zonal (**a**) and meridional (**b**) components, as well as the ratio percentage of the eddy heat flux to the mean flow flux (**c**). The maps in Fig. 5 are generated by using the Matlab software (http://www.mathworks.com/products/matlab/).

The heat storage in the upper mixed-layer can be defined as ${\rm{Q}}={{\rm{\rho }}C}_{{\rm{p}}}{{\rm{H}}}_{{\rm{s}}}{\rm{T}}$^26^; with this definition, the heat flux by the mean flow is calculated by ${F}_{Q\_mean}^{x}=\rho {C}_{p}{H}_{s}Tu$ and ${F}_{Q\_mean}^{y}=\rho {C}_{p}{H}_{s}Tv$, where *u* and *v* represent the mean velocity components. Fig. 5 shows the heat flux of the mean flow in the zonal (a) and meridional (b) directions. It reveals that to west of Taiwan and Luzon Island, there are very high heat fluxes caused by the strong Kuroshio Current, which transports the heat northwestward east of Luzon Island and northeastward east of Taiwan. A southward heat flux is seen east of Vietnam, associated with the western boundary current of the SCS. The Kuroshio Current-caused heat flux is as high as ~2.2 × 10^9^ W m^−1^ (maximum value); in most of the SCS, however, the mean flow heat flux is much less, ~4.9 × 10^8^ W m^−1^ (maximum value) in the northern SCS, and 9.3 × 10^8^ W m^−1^ (maximum value) east of Vietnam. In the northern SCS, the eddy heat transport is as high as ~3.4 × 10^7^ W m^−1^ (maximum value), about 27.0% (maximum value) of the mean flow heat transport. However, southeast of Vietnam on the west side of the SCS, the eddy heat flux can reach 4.1 × 10^7^ W m^−1^ (maximum value), while the mean flow-caused heat flux drops to ~3.0 × 10^7^ W m^−1^, and the eddy heat flux reaches ~40% of the mean flow heat flux. The eddy heat flux is northward, opposed to the mean flow flux (southward) southeast of Vietnam. Therefore, although the mean flow-caused heat transport is higher, the eddy heat flux may play an important role in the local heat balance in the SCS. The ratio (percentage) of the eddy heat flux to the mean flow flux is calculated in the SCS (Fig. 5c). A high percentage is found southeast of Vietnam and in the northern SCS (~40%), and in some locations (such as southeast coastal waters of China mainland), this percentage is higher than 60%.

**Figure 6:**
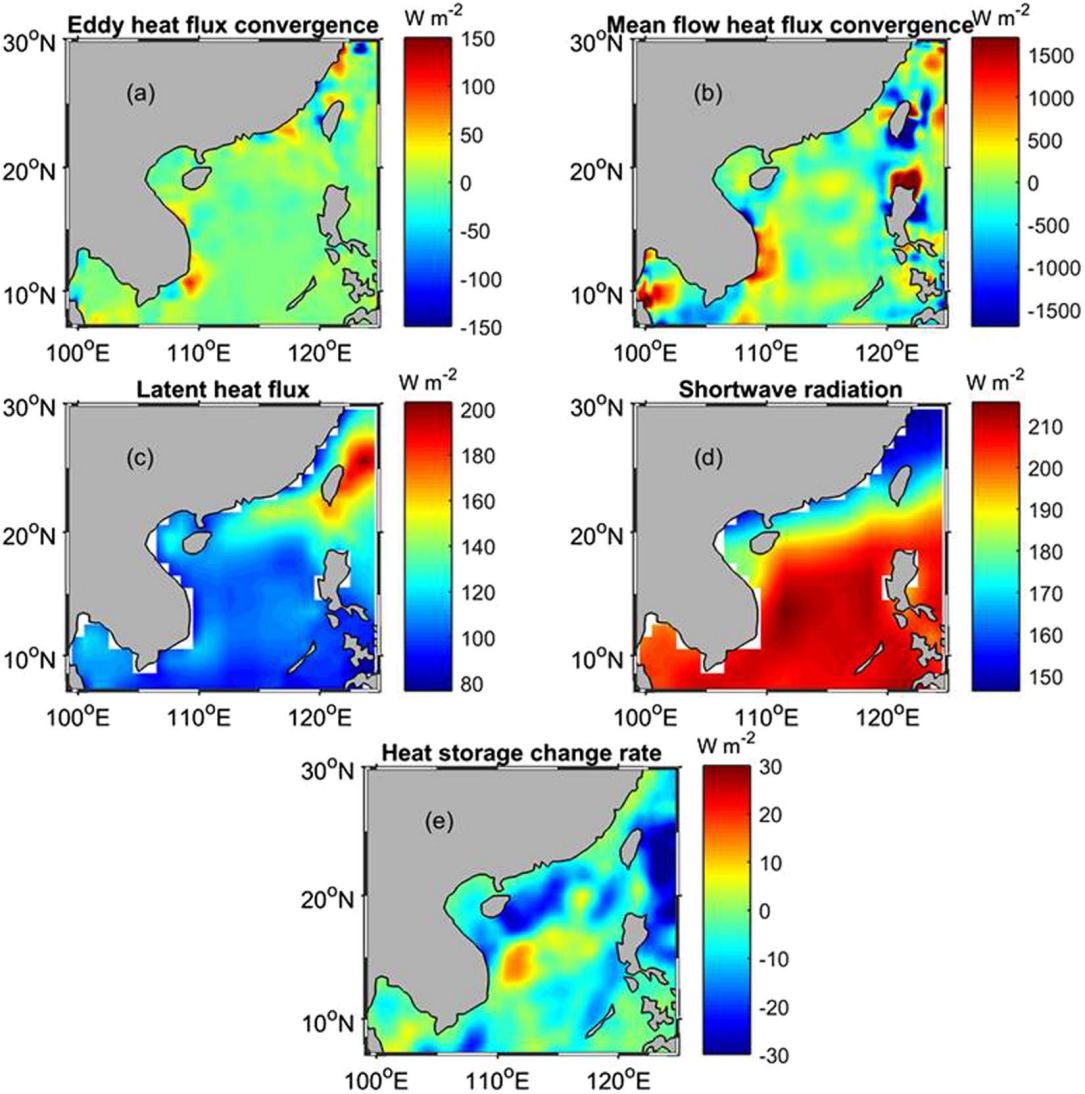
The long-term means of eddy heat flux convergence (**a**), mean flow heat flux convergence (**b**), latent heat flux (**c**), shortwave radiation (**d**), and the heat storage change rate (**e**). The map in Fig. 6 is generated by using the Matlab software (http://www.mathworks.com/products/matlab/).

It is obvious that heat transport is not conserved in the SCS, and therefore, divergence and convergence of the heat energy may occur. The heat flux convergence can be calculated by

7 $$C=-(\frac{\partial {F}_{Q}^{x}}{\partial x}+\frac{\partial {F}_{Q}^{y}}{\partial y}),$$

where *C* represents the heat convergence that reflects accumulation (positive *C*) or dispersion (negative *C*) of the heat flux at a certain location. The convergence of the long-term eddy heat fluxes is derived, as shown in Fig. 6a. A maximum value of 101.0 W m^−2^ is seen at 10.68 °N, 109.20 °E southeast of Vietnam. Positive values are also found in the northern SCS, and the typical convergence value is ~20.0 W m^−2^. The convergence of the heat flux caused by the mean flow is also derived and shown in Fig. 6b. Associated with the heat flux of the strong Kuroshio Current, regions of strong convergence and divergence in the mean flow flux are seen east of Taiwan and Luzon Islands, and a high heat convergence region is also found in the coastal waters of southeast Vietnam. The eddy heat convergence can be as high as 101.0 W m^−2^ southeast of the Vietnam coastal waters, and around this maximum eddy heat convergence area, the mean flow heat convergence drops to ~500 W m^−2^, on the same order as the eddy heat convergence, indicating that the eddy heat convergence could influence the heat budget balance in this region.

Latent heat fluxes and solar shortwave radiation also play an important role in the ocean upper layer heat budget. Fig. 6c,d show the long-term means (for January 2006~December 2009 as the shortwave radiation in 2010 is not provided) of the latent heat flux and shortwave radiation, respectively. The latent heat flux and shortwave radiation data are obtained from the global air-sea flux fields of Objectively Analyzed air-sea Fluxes (OAFlux) project of the Woods Hole Oceanographic Institution, (http://oaflux.whoi.edu/index.html)^24,27^. In addition, the heat storage change rate is derived by dQ/dt, as illustrated in Fig. 6e for the long-term mean (in December 2005 ~November 2010).

The maximum heat storage change is observed east of Vietnam on the west side of the central SCS, and the heat storage decreases in the northern SCS. Consistent with the convergence of the eddy heat flux, the latent heat flux also shows a high magnitude region southeast of Vietnam, and another high magnitude region in the northern SCS. Although the mean flow heat convergence is much higher than the eddy heat convergence, Fig. 6c reveals that the latent heat can be more spatially correlated with the eddy heat convergence than the mean flow convergence. The maximum value of shortwave radiation is seen on the west side of the central SCS east of Vietnam, corresponding to the local maximum heat storage increase and the high heat convergence of the mean flow. By comparing different heat sinks and sources, it is suggested that the eddy heat convergence may be related to the high latent heat flux appearing southeast of Vietnam and that the strong shortwave radiation and high heat convergence of the mean flow on the west side of the central SCS can be responsible for the heat storage increase in the area.

**Figure 7:**
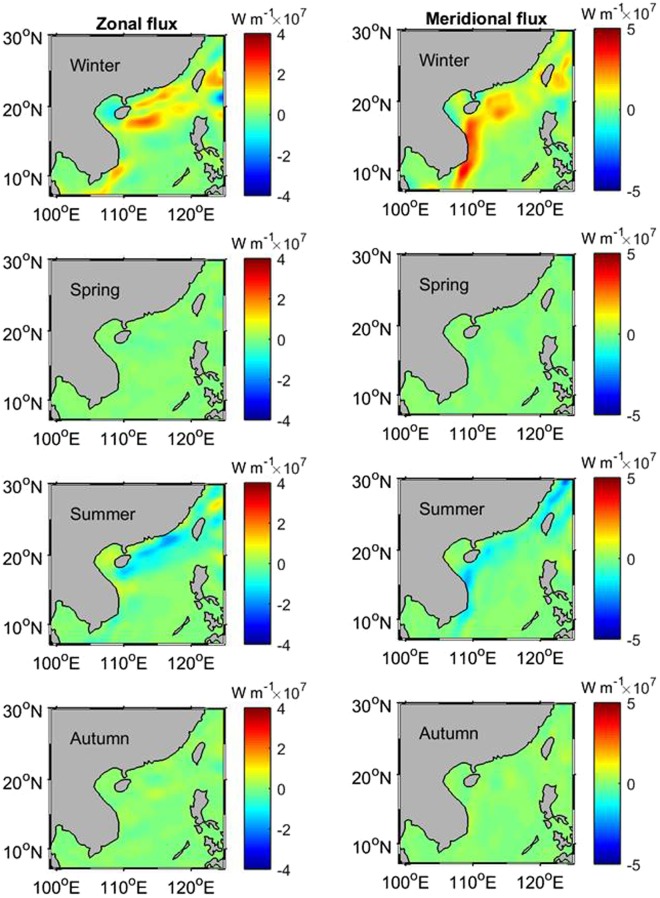
The seasonal means of the heat flux caused by the covariance between the mixed-layer depth and the eddy heat flux in the zonal (left panel) and meridional (right panel) directions. The map in Fig. 7 is generated by using the Matlab software (http://www.mathworks.com/products/matlab/).

For simplicity, the mixed-layer depth used in this study is the value of the seasonal mean, and therefore, the covariance between the mixed-layer depth and the eddy heat flux is not accounted for in the calculation with Equations 5 and 6. Herein, the covariance between the mixed-layer depth and the eddy heat flux is evaluated by ${F}_{Q\_corr}^{x}=\rho {C}_{p}\overline{H^{\prime} T^{\prime} u^{\prime} }$ and ${F}_{Q\_corr}^{y}=\rho {C}_{p}\overline{H^{\prime} T^{\prime} v^{\prime} }$, where *H*′ represents the mixed-layer depth fluctuation relative to the seasonal mean. The covariance fluxes in the four seasons are then calculated and displayed in Fig. 7, which reveals that the covariance flux has a similar pattern to the eddy flux (Fig. 2) in winter with a weaker intensity (2~3 × 10^7^ W m^−1^), while in other seasons, the covariance flux is an order of magnitude less. The covariance flux can enhance the eddy flux in winter, but in the other seasons, it cannot significantly affect the eddy heat flux.

## Conclusions

In this study, the horizontal eddy heat flux is calculated for the upper mixed-layer in the South China Sea based on the satellite observed sea surface height and temperature, as well as the reanalysis mixed-layer depth. The calculation results indicate that the long-term flux has distinct spatial characteristics in the SCS, and is comparable with the strong eddy flux region such as the Kuroshio extension. The eddy fluxes show a northward transport of the heat on the west side of the SCS from the tropical ocean to mid-latitudes; the heat flows into the southern side of the SCS through the Java Sea and out through the northern Luzon Strait. The eddy heat flux has a prominent semi-annual cycle, strongest in winter and summer and weakest in spring and autumn. In winter and summer, the eddy flux is as high as ~5.0 × 10^7^ W m^−1^ for the upper mixed-layer, and heat can flow into the East China Sea through the Taiwan Strait, which may affect the temperature along the coast of southeast China. The convergence of the heat flux reveals that heat accumulates in the northwestern SCS and the coastal area southeast of Vietnam. Comparison among the eddy heat flux convergence, the heat flux convergence of the mean flow, the latent heat flux, shortwave radiance, and the heat storage change in the upper mixed-layer suggests that the eddy heat flux may be related to the heat storage increase southeast of Vietnam. On the west side of the central SCS, the heat storage increase may be connected with the shortwave radiation and the mean flow heat flux convergence. Our study suggests that in the SCS, the eddy heat flux can reach more than 60% of the mean flow heat transport, and therefore, it should be well accounted for to accurately model heat structure and climate impacts to the region.
